# Case selection for urological input in planned laparoscopic rectovaginal endometriosis surgery

**Published:** 2019-06

**Authors:** G Fisher, RD Smith, E Saridogan, A Vashisht, S Allen, V Arumuham, A Cutner

**Affiliations:** Endometriosis Unit, Department of Women’s Health, University College Hospital London, 235 Euston Rd, London, NW1 2BU, United Kingdom;; Department of Endoluminal Endourology, Institute of Urology, University College Hospital London, 16-18 Westmoreland Street, London, W1G 8PH, United Kingdom.

**Keywords:** endometriosis, laparoscopy, urology, ureter

## Abstract

**Background:**

Surgery for deep endometriosis often requires input from urological surgeons. This study aims to determine pre-operative and intra-operative factors that influence the need for urological input in laparoscopic resection of rectovaginal endometriosis and to assess the usefulness of a scoring system to predict this.

**Methods:**

We conducted a retrospective cohort study of 230 patients undergoing laparoscopic excision of deep endometriosis, at a tertiary referral centre for endometriosis in London UK, 2011 to 2015. Data from pre-operative assessment, surgery and post-operative follow up were analysed and patients were categorised according to their pre-operative and intra-operative risk factors. The primary outcome measure was the requirement of intra-operative input by urological surgeons.

**Results:**

The median age was 35 years. In addition to the excision of endometriosis, 19.6% patients (45 patients) underwent hysterectomy, 14.8% (34 patients) required JJ stent placement, 6.1% (14 patients) had bowel resections and 2.6% (6 patients) required an ileostomy. 93.9% (216 patients) were considered normal-risk pre-operatively, of whom 89.4% (193/216) did not require any intra-operative urological input. 10.6% of this normal-risk group (23/216) required JJ stents, of whom 69.6% (16/23) also required a hysterectomy or bowel resection. Post operative complications occurred in 0.9% (2/216) of normal-risk patients, with none having required intra-operative urological reconstruction.

Six percent (14 patients) were deemed to be increased-risk pre-operatively, of whom 78.6% (11/14) required JJ stent insertion. Thirty-six percent of increased-risk patients (5/14) had pre-operative renal dysfunction demonstrated on MAG3/DMSA and 80.0% of these (4/5) required intra-operative ureteric reconstruction.

**Conclusions:**

Patients considered normal-risk pre-operatively, planned for excision, without hysterectomy or bowel resection, can be safely managed without specific urology input. Patients with risk-features are highly likely to require urological input, particularly for JJ stent insertion. Patients with pre-operative renal dysfunction, demonstrated on MAG3/DMSA, have a high chance of requiring intra-operative ureteric reconstruction and are best managed with pre-planned reconstructive urologist input.

## Introduction

Deep endometriosis (DE) is the disease in its most severe form. It occurs in 20% of women with endometriosis (Koninckx et al., 2012; Bazot and Dara, 2017). Its definition is commonly accepted to be where there are implants of endometrial tissue found beneath the peritoneum, to a depth of 5mm or more (Koninckx et al., 2012; Nisenblat et al., 2016). This is most commonly found in the posterior pelvic compartment and can involve the rectovaginal septum, the uterosacral ligaments, the vagina, ureters and the lower part of the colon (Chapron et al., 2003; Nisenblat et al., 2016; National Centre for Health and Care Excellence [NICE], 2017; Gordts et al., 2017).

Medical treatment can be used to manage endometriosis with good effect (Vercellini et al., 2009). However, DE often requires surgical resection of lesions. Due to the locations of endometriosis, this resection can include bowel surgery, with shaving, disc resection, or segmental resection (Ford et al., 2004). Where there is involvement of the ureter, either directly, or indirectly (through scarring and distortion of the normal anatomy), input by urological surgeons is often required for complete and safe resection of the disease.

NICE recommend that centres providing specialist care for endometriosis should exist as multidisciplinary teams and include urological surgeons as part of that set up (NICE, 2017). The British Society for Gynaecological Endoscopy (BSGE) instruct further regulation for Endometriosis Centres, requiring a sufficient workload of complex cases, with resection of rectovaginal lesions of endometriosis involving dissection of the pararectal space and requiring these centres to have a named colorectal surgeon and supporting urological surgeons as part of their teams (BSGE, 2018).

Surgery in gynaecology is associated with injuries to the ureter. This is due to the close association of the ureter with structures involved in these surgeries. The rates of injury range from 0.2% to 2.2% for gynaecological laparoscopy in general (Ostrzenski et al., 2003; Brandes et al., 2004; Cicco et al., 2007, 2008; Park et al., 2012; Alves et al., 2017;). The rate for procedures for deep endometriosis are reported as up to 1.5% (Donnez et al., 2002; Park et al., 2012; Byrne et al., 2018; Smith and Cutner, 2018).

A recent large, multi-centre retrospective review of BSGE Centres (Byrne et al., 2018), showed that JJ stents were required in 9.2% of procedures and ureteric nodules were removed in 9.0% of procedures. Though uncommon, ureteral endometriosis can rarely require resection of portions of the ureter with re-implantation (Donnez et al., 2002).

The risk factors for the requirement of urological input in DE surgery have not been clearly defined. The majority of procedures will not require the assistance of urological surgeons. In order to achieve high quality Multi Disciplinary Team planning and perform safe surgery, it is important to select the correct patients for the availability or attendance of urological surgeons prior to operating. Smith and Cutner (2018) suggested a scoring system, shown in Table I, which could be used to guide this selection.

**Table I:** — Risk scoring as suggested by Smith & Cutner (2018).

0 - No concerns
1 - Previous surgery involving ureteric dissection
2 - Loin pain
3 - Hydronephrosis
4 - JJ stent in-situ
5 - Loss of function on renography (MAG3 or DMSA)

This scoring system evaluates a number of pre-operative presenting features, which may be related to ureteric involvement by endometriosis and tries to guide which cases are more likely to require input by urologists intra-operatively. Smith and Cutner (2018) recommend that women with pre-operatively identified hydronephrosis should have pre-operative review by urological surgeons for operative planning and consideration of JJ stent insertion prior to the procedure. These women should also have pre-operative isotope renography by MAG3 to accurately assess for obstruction and/or loss of function. In addition, they suggest that, by assessing women in this way and utilising their pre-operative scoring system, women at high risk of ureteric involvement of disease can be identified pre-operatively. Therefore, steps can be taken to reduce the risk of injuries to the ureter and improve outcomes for these high risk women.

We carried out an audit of the surgical management of recto-vaginal endometriosis in our unit with aims to assess the usefulness of this scoring system and to further identify those pre-operative and intra-operative risk factors that are associated with a need for intra- operative urological input.

## Materials and methods

All patients undergoing planned laparoscopic excision of deep endometriosis (involving dissection of the pararectal space) at University College London Hospital (UCLH), between January 1 2011 and December 31 2015, were included in this retrospective review. Eligible cases were identified from the BSGE database. Relevant pre-operative, intra-operative and post-operative data were retrieved from the medical notes. Information on age at operation, previous pelvic surgery, previous ureteric dissection, presence of loin pain and pre-operative insertion of JJ stents or nephrostomies were obtained from clinic letters and operation notes. Evidence of hydronephrosis was assessed using pre-operative imaging and reports. Pre-operative evidence of reduced relative function of an affected kidney (henceforth referred to as “loss of renal function”) was identified from MAG3 or DMSA data. Intra-operative urological issues were assessed from a detailed review of surgical operation notes. Post- operative problems were identified through the need for post-operative JJ or nephrostomy tubes or the need for post-operative urological reconstruction.

We utilised the pre-operative scoring system, as described by Smith and Cutner (2018) to assess for urological risk. Patients were scored according to their highest scoring factor in each domain, as summarised in Table I. For example, a patient with a JJ stent in-situ would score 4, whereas if there were JJ stent in-situ and MAG3/DMSA evidence of renal dysfunction then the score would be 5. We assessed the requirement for intra-operative urological input for normal-risk patients (with a pre-operative score 0) and higher risk patients (score >=1) and for the risk of post-operative urological complications in both groups.

The surgical treatment and follow-up were part of standard management of women attending The Endometriosis Centre at UCLH. This is a large, BSGE accredited endometriosis centre, in central London. All patients were reviewed pre-operatively in the Endometriosis Centre and at least once 3 months post-operatively (or earlier if required). Further data were collected at 6, 12 and 24 months, as per BSGE Endometriosis Centres protocol. In our unit, urological input was usually required for the following reasons; endourological input for patients who were pre-operatively found to have hydroureteronephrosis with or without loss of renal function, or for those with extensive intra-operative dissection around the ureter and concern about ischaemic or thermal damage to the ureter (for JJ stent insertion). Reconstructive urology input was arranged for patients who had severe ureteric stricture which required reimplantation of the ureter. JJ stents were placed in order to protect the ureter with the intention of minimizing the risk of post-operative uretreric stricture formation. Patients who experienced post-operative ureteric complications were also seen by the endourology or reconstructive urology teams, as indicated.

This retrospective review of anonymised data was performed following the ethical principles found in the Declaration of Helsinki, as developed by the World Medical Association. This audit had the approval of the hospital Audit Department (Institutional Review Board approval).

## Results

### 

Over the study period, a total of 230 women underwent planned laparoscopic treatment of deep endometriosis at our centre. The median age at operation was 35 years (range 20-52 years).

We were unable to accurately assess for previous ureteric dissection as the majority of women were referred from other centres and specific information regarding previous operation findings was lacking. The overall pre-operative assessment demonstrated that 3.5% (8/230) of the patients reported loin pain, 3.9% (9/230) had hydronephrosis, 1.7% (4/230) had pre-existing unilateral JJ stents in-situ, 0.5% (1/230) had a unilateral nephrostomy in-situ due to obstruction of the ureter and 2.2% (5/230) had evidence of loss of renal function on MAG3 or DMSA. The resulting groups by risk score can be seen in Table II when assessed by the scoring system described in our Methods.

**Table II:** — Numbers of women by score.

Pre-op score	Number of patients affected	Percentage of entire cohort
0 - No concerns	216	94%
1 - Previous ueteric dissection	Not assessed	N/A
2 - Loin pain	4	1.7%
3 - Hydronephrosis	3	1.3%
4 - JJ Stents in-situ	2	0.9%
5 - Pre-existing renal dysfunction on MAG3/DMSA	5	2.2%

All 230 women underwent excision of recto-vaginal endometriosis as planned. In addition, 19.6% (45/230) underwent hysterectomy, 6.1 % (14/230) also had a bowel resection and 2.6% (6/230) required a defunctioning ileostomy as part of their surgical management. Some patients had a combination of these additional procedures. From a urological perspective, 14.8% of patients overall (34/230) had JJ stents inserted. 2.6% of patients overall (6/230) needed intra-operative ureteric surgery: 0.4% (1/230) had an incision to the ureter and 0.4% (1/230) had a transection of the ureter; both were recognised and repaired intra-operatively. 1.7% (4/230) required intra-operative ureteric reconstructions, of which two were planned pre-operatively and two were unplanned. All four of the patients requiring reconstruction were “increased-risk” cases.

### Patients with normal pre-operative risk

Of the cohort, 93.9% (216/230) were regarded as normal-risk, i.e. without any pre- operative Urological risk factors. 89.4% (193/216) of these “normal-risk” patients did not require any urological input in their procedures.

### Intra-operative urological input

10.6% (23/216) of the normal-risk patients underwent a significant dissection of the ureter necessitating the insertion of JJ stents at the time of their endometriosis surgery. Among normal-risk patients, 55 women underwent operations involving more complex procedures: either hysterectomy or bowel resection. Normal-risk women, undergoing such procedures, had a 29.1% (16/55) chance of requiring JJ stent insertion as part of their procedure. Conversely, patients from the normal-pre-operative- risk group who did not require hysterectomy or bowel surgery had a 4.3% likelihood of requiring a JJ stent (7/161). Overall, 70% of the JJ stent insertions occurring in normal-risk women (16/23) occurred in patients undergoing endometriosis surgery that also involved hysterectomy or bowel resection. It is important to note that the insertions of these stents were not pre-planned. They were deemed necessary intra-operatively due to the extent of the dissection around the ureters and the possible risk of compromise secondary to ischaemic or thermal damage. No normal-risk patients required intra-operative ureteric reconstructions.

### Post-operative urological input

Only two of the patients categorised, pre-operatively, as normal-risk and who underwent intra-operative JJ stent insertion, required additional post-operative urological intervention. The first patient underwent a hysterectomy and bilateral Salpingo-oophorectomy in addition to bilateral ureterolysis including excision of a recto-vaginal nodule. Bilateral JJ stents were placed at the end of the procedure. These were planned for removal with bilateral retrograde studies three months later. The retrograde study on the left caused extravasation of contrast from the upper pole calyx, requiring the left sided stent to be replaced. The right sided JJ stent was also replaced. The patient returned for bilateral retrograde studies after a further three months, at which stage the findings were reassuring, and both stents were removed. A follow-up MAG3 renogram showed that the relative function of her two kidneys were within normal limits (Right 44% / Left 56%), both showing a “dilated unobstructed” picture on the drainage phase. No further urological intervention has been required.

The second patient had a laparoscopic shaving of a rectal endometriotic nodule and a right sided JJ stent placed intra-operatively, which was removed approximately six weeks later. Following this, she developed a urine leak per vaginam, for which a CT urogram was arranged that showed urine leakage into the pelvis and confirmed a right uretero-vaginal fistula. She underwent a “Rendezvous” procedure for this, involving a ureteroscopy and antegrade wire insertion via a percutaneous nephrostomy. This restored ureteric continuity, allowing a JJ stent to be re-inserted, A subsequent ureteroscopic assessment of the ureter showed a satisfactory lumen, and healing of the uretero-vaginal fistula, such that the JJ stent was removed. She has been followed up by isotope renography, and has maintained adequate function (the affected kidney contributes 41% of her overall renal function) with a dilated, unobstructed pattern on the MAG 3 renogram.

Two further normal-risk patients (who had not required intra-operative urological input) had post-operative urological problems, representing 0.93% of the normal-risk cohort (2/216) and 0.87% of the total patient group (2/230). One patient presented with loin pain 6 weeks post-operatively, and had hydronephrosis demonstrated on renal ultrasound. This was treated by a JJ stent insertion for 3 months, with no further sequelae after the stent was removed. The other patient had a rectal perforation presenting at 5 days, and required a laparotomy and defunctioning ileostomy. A post-operative JJ stent insertion was required for hydronephrosis, which did not resolve. The patient required a subsequent reconstruction with ureteric reimplantation.

### Patients with increased pre-operative risk

6.1% of the women in our series (14/230) had identifiable pre-operative risk factors, according to Table I, for requiring urological input. 4 women had loin pain only (giving a pre-operative score of 2); 3 women had hydronephrosis without any higher-risk features (giving a pre-operative score of 3), 2 women had pre-exisiting JJ stents with no higher-risk feature (giving a pre-operative score of 4) and 5 women had established loss of ipsilateral renal function, demonstrated on MAG3 or DMSA (a pre-operative score of 5), one of whom one had pre-operative nephrostomies.

Of these increased-risk women, 78.6% (11/14) (required JJ stent insertion intra- operatively. Of the 4 women with loin pain and no hydronephrosis, 3 (75%) required intra-operative JJ stents. Of the 5 women with pre-operative hydronephsosis or pre-operative JJ stents but no loss of renal function, 4 (80%) required intra-operative JJ stent insertion or exchange. None of these patients required ureteric re-implantation.

Of this increased-risk group, 28.6% (4/14) required intra-operative ureteric reconstruction. Two had planned ureteric reconstructions and 2 were found to have ureteric involvement of the disease, and injuries to the ureter during resection of nodules, necessitating reimplantation of the ureter. All four of these patients who required reconstruction were derived from the group with pre-operative evidence of loss of renal function on MAG3/DMSA. This equates to an 80% likelihood (4/5 patients) of intra-operative ureteric re-implantation in patients with established loss of renal function secondary to endometriosis.

No increased-risk patients required post-operative re-implantations.

### Intra-operative risk factors

In addition to the pre-operative risk factors detailed above, the need for a hysterectomy or bowel surgery as part of the endometriosis treatment also increased the likelihood of urological sequelae, both for normal-risk and high risk patients. Table III shows the consequences that hysterectomy / bowel surgery had to normal and increased-risk patients. Normal-risk patients, undergoing excision of rectovaginal endometriosis but not requiring hysterectomy or bowel surgery required urological intervention in 4.3% (7/161) of cases. When normal -risk patients required these additional procedures the rate of urological intervention increased to 29.1% (16/55). All interventions in low risk patients were the insertion of JJ stents. No reconstructions were required in the low risk group. Increased -risk patients, not requiring hysterectomy or bowel surgery required urological intervention in 72.7% (8/11). Of these 8 cases requiring intervention, 5 required JJ stent placement and 3 required ureteric reconstruction. Of increased-risk patients, undergoing procedures including hysterectomy or bowel surgery, 100% (3/3) required urological intervention. This included 2 insertions of JJ stents and 1 ureteric reconstruction.

**Table III:** — Risk of urological intervention by pre and intra operative risk factors.

Patient risk/Surgery type	Number of cases	Intervention & nature	Intervention rate
Normal-risk – No bowel surgery/hysterectomy	161	7 (all stents)	4.3%
Normal-risk - With bowel surgery/hysterectomy	55	16 (all stents)	29.1%
Increased-risk – No bowel surgery/hysterectomy	11	8 (5 stents/3 reconstructions)	72.7%
Increased-risk – With bowel surgery/hysterectomy	3	3 (2 stents/1 reconstruction)	100%

## Discussion

To our knowledge, this is the first paper that has attempted to guide Multi-Disciplinary Team (MDT) planning for urology input at rectovaginal endometriosis surgery. Whilst the concept of a pelvic endometriosis surgeon has been suggested by some specialists, the level of expertise in a variety of disciplines would be extensive. Thus, in the majority of units the concept of multidisciplinary working is accepted. This is in line with current UK practice.

Overall, injuries to the ureter in gynaecological laparoscopy occur in 0.2-2.2% of all benign procedures (Ostrzenski et al., 2003; Brandes et al., 2004; Cicco et al., 2007, 2008; Park et al., 2012; Alves et al., 2017). Established risk factors for ureteric injury include: hysterectomy, endometriosis, previous pelvic surgery, pelvic inflammatory disease, history of pelvic radiation and congenital anomalies (Brandes et al., 2004, Weingertner et al., 2008; Park et al., 2012). In this study, all women had deep endometriosis, with recto-vaginal disease and also included women with pre-operative ureteric involvement with endometriosis. The overall incidence of ureteric compromise (unintentional incision or transection of the ureter, intra-operative ureteric reconstruction, post-op insertion or replacement of JJ stents/nephrostomy, post-op reimplantation) was 2.6% (6/230). Higher rates of injury in our cohort are not surprising, given that 38% of ureteric injuries, sustained during gynaecological laparoscopy, occur in patients with endometriosis (Weingertner et al., 2008) and that rates of hydronephrosis are significantly higher in patients with rectovaginal endometriosis and ureteric injuries are more common in patients with this finding (Alves et al., 2017). The overall rate of JJ stent insertion among our cohort of 14.2% is higher than that of the BSGE cohort (Byrne et al., 2018), at 9.2%, which may reflect the complexity of our case-mix.

Patients pre-operatively identified as normal-risk required urological input in 10.6% of cases (23/216). All 23 of these examples of urological input were accounted for by the insertion of intra-operative JJ stents, the majority of which occurred in patients undergoing hysterectomy or bowel resection at the time of their endometriosis surgery. This increased-risk among these more complex procedures is supported in the literature, which states that bowel surgery increases the risks of endometriosis surgery significantly, with major complications. These include rectovaginal fistulae, ureteral fistulae, pelvic abscess, bleeding requiring re-intervention, ureteral stenosis, vaginal apex necrosis, vesicovaginal fistulae, haematoma formation, anastomotic leaks and stenosis of ileostomy (Mohr et al., 2005; Kondo et al., 2011). These complications occur in 24-38% of operations involving segmental resections and 18-23% of operations involving disc resections (Mohr et al., 2005; Kondo et al., 2011).

The normal-risk patients generally did well post-operatively. 1.9% (4/216) had a Clavien grade III complication. There were three patients successfully managed with endourology (2 were managed with JJ stents alone, one with JJ stent and rendezvous procedure) and 1 (0.5%) requiring a secondary procedure for a ureteric reconstruction.

This complication may have been predictable, given the patient developed a bowel leak and peritonitis, in the presence of a recent ureteric dissection. Prophylactic stenting of the ureters, on diagnosis of the bowel perforation, may well have avoided the need for the secondary urological procedure. Our data suggests that normal-risk patients, who are not anticipated to require hysterectomy or bowel resection, have infrequent need for urological input. In the cases that urological input is required, it is generally the prophylactic insertion of JJ stents which can usually be straightforwardly requested through an on-call urology service. It therefore seems reasonable not to arrange specific urological presence in theatre for these cases, although notifying the on-call team prior to the start of the case would be good practice.

Patients with a pre-operative normal-risk status who undergo hysterectomy or bowel resection should be considered at higher intra-operative risk for ureteric involvement and therefore urological availability, for the potential need of insertion of JJ stents, should be planned pre-operatively. No intra-operative ureteric reconstructions were required for normal-risk patients and therefore pre-planned availability for reconstructive urological surgery does not seem necessary in this group.

Pre-operative loin pain and hydronephrosis were confirmed to be risk factors for requiring an intra-operative JJ stent, although none of the patients with these pre-operative risk factors required reimplantation. Re-implantation at the time of surgery was only required where there was evidence of pre-operative loss of ipsilateral renal function, as demonstrated on MAG3 or DMSA, and was needed in 4 of the 5 patients with this pre-operative factor. Our data suggests that the pre-planned availability of a reconstructive urologist is only necessary in patients with confirmed pre-operative loss of renal function.

We were unable to assess the full scoring system, as described by Smith and Cutner (2018). The limited availability of operation notes from other centres made it impossible to assess the impact of previous ureteric dissection on the risk for urological input. However, it is likely to be a risk factor and we would, therefore, recommend further assessment of this.

## Conclusions

NICE recommends multidisciplinary management of women with recto-vaginal disease, with teams including urological surgeons (NICE, 2017). Our data demonstrates that, in recto-vaginal endometriosis, the scoring system described by Smith and Cutner (2018) helps to predict both the need for, and type of intra-operative urological input. Anticipating the risk of ureteric injury and guiding the availability of urological surgeons for stent insertion or reconstruction should help with service planning when managing women with deep endometriosis, and specifically those cases where an endo-luminal urologist is required and those cases where the availability of reconstructive urology should be available.

Normal-risk patients, who are not planned for hysterectomy or bowel resection, do not require pre-arranged urological input. When either a hysterectomy or bowel resection is required, intra-operative JJ stent insertion is increasingly likely. Increased-risk patients without loss of ipsilateral renal function on MAG3/DMSA frequently require insertion of JJ stents but do not require re-implantation. Pre-operative arrangements should therefore be made to facilitate stent insertion (i.e a radiolucent operating table suitable for fluoroscopy and a urologist and radiographer on “stand by”). Patients with pre-existing loss of ipsilateral renal function on MAG3/DMSA are at high risk of requiring ureteric reconstruction and planning for these cases should include the scheduled availability of a reconstructive urologist.

## References

[1] Alves J, Puga M, Fernandes R (2017). Laparoscopic Management of Ureteral Endometriosis and Hydronephrosis Associated With Endometriosis.. J Minim Invasive Gynecol.

[2] Bazot M, Dara E (2017). Diagnosis of deep endometriosis: clinical examination, ultrasonography, magnetic resonance imaging, and other techniques.. Fertil Steril.

[3] Brandes S, Coburn M, Armenakas N, McAninch J (2004). Diagnosis and management of ureteric injury: An evidence-based analysis.. BJU Int.

[4] Requirements to be a BSGE Accredited Centre. https://www.bsge.org.uk/requirements-to-be-a-bsge-accredited-centre/.

[5] Byrne D, Curnow T, Smith P (2018). Laparoscopic excision of deep rectovaginal endometriosis in BSGE endometriosis centres: a multicentre prospective cohort study.. BMJ Open.

[6] Chapron C, Fauconnier A, Vieira M (2003). Anatomical distribution of deeply infiltrating endometriosis: Surgical implications and proposition for a classification.. Hum Reprod.

[7] Cicco CD, Dávalos MLR, Cleynenbreugel BV (2007). J Minim Invasive Gynecol.. Journal of Minimally Invasive Gynecology.

[8] Cicco CD, Schonman R, Craessaerts M (2009). Laparoscopic management of ureteral lesions in gynecology.. Fertil Steril.

[9] Donnez J, Nisolle M, Squifflet J (2002). Ureteral endometriosis: A complication of rectovaginal endometri- otic (adenomyotic) nodules.. Fertil Steril.

[10] Ford J, English J, Miles WA (2004). Pain, quality of life and complications following the radical resection of rectovaginal endometriosis.. BJOG.

[11] Gordts S, Koninckx P, Brosens I (2017). Pathogenesis of deep endometriosis. Fertil Steril.

[12] Kondo W, Bourdel N, Tamburro S (2011). Complications after surgery for deeply infiltrating pelvic endometriosis.. BJOG.

[13] Koninckx PR, Ussia A, Adamyan L (2012). Deep endometriosis: Definition, diagnosis, and treatment.. Fertil Steril.

[14] Mohr C, Nezhat FR, Nezhat CH (2005). Fertility considerations in laparoscopic treatment of infiltrative bowel endometriosis.. JSLS.

[15] (2017). Endometriosis: diagnosis and management. NICE guideline [NG73].

[16] Nisenblat V, Bossuyt PM, Farquhar C (2016 Feb 26). Imaging modalities for the non-invasive diagnosis of endometriosis. Cochrane Database Syst Rev.

[17] Ostrzenski A, Radolinski B, Ostrzenska KM (2003). A Review of Laparoscopic Ureteral Injury in Pelvic Surgery.. Obstet Gynecol Surv.

[18] Park JH, Park JW, Song K (2012). Ureteral injury in gynecologic surgery: A 5-year review in a community hospital.. Korean J Urol.

[19] Smith RD, Cutner A (2018). Endometriosis and the ureter. J Endoluminal Endourology.

[20] Vercellini P, Crosignani PG, Somigliana E (2009). Hum Reprod. Medical treatment for rectovaginal endometriosis: What is the evidence.

[21] Weingertner AS, Rodriguez B, Ziane A (2008). The use of JJ stent in the management of deep endometriosis lesion, affecting or potentially affecting the ureter: A review of our practice.. BJOG.

